# The effect of hydrochlorothiazide on the recurrence of nonmelanoma skin cancer: a 7-year retrospective study comprising 300 patients

**DOI:** 10.55730/1300-0144.5638

**Published:** 2023-01-04

**Authors:** Burak ÖZKAN, Süleyman SAVRAN, Kadri AKINCI, Abbas ALBAYATİ, Pınar İNCEL UYSAL, Çağrı Ahmet UYSAL

**Affiliations:** 1 Department of Plastic Surgery, Başkent University Hospital and Faculty of Medicine, Ankara, Turkey; 2 Department of Plastic Surgery, Yalova State Hospital, Yalova, Turkey; 3 Deparment of Dermatology, TOBB ETU Hospital, Ankara, Turkey

**Keywords:** Cancer, hydrochlorothiazide, recurrence, skin

## Abstract

**Background/aim:**

Hydrochlorothiazide (HCTZ) possesses well-described photosensitizing properties, and a causal association with nonmelanoma skin cancer (NMSC) was recently shown. However, previous studies have not shown whether HCTZ use is associated with the risk of recurrence of basal cell carcinoma (BCC) or squamous cell carcinoma (SCC). This study aims to investigate the association between HCTZ use and recurrence in patients with NMSC.

**Materials and methods:**

We identified cases with NMSC from our hospital archives during the period between 2013 and 2019. Patients were divided into groups according to the pathological diagnosis, HCTZ use, and recurrence. Multivariable analysis was performed to determine factors associated with recurrence in BCC and SCC.

**Results:**

Recurrences of BCC were significantly higher in HCTZ users with ORs of 4.839221 (95% confidence interval [CI], 1.22–19.12). In HCTZ users, NMSC cases were associated with increased age (p < 0.001 for both BCC and SCC). BCC recurrences were statistically significant with age, longer follow-up, and positive margins after excision in HCTZ users (p = 0.048, 0.020, and, 0.003, respectively). SCC recurrences were not significantly associated with HCTZ use.

**Conclusion:**

HCTZ use is significantly associated with BCC recurrences. Especially in the elderly population, cases with a positive margin should be followed closely.

## 1. Introduction

Nonmelanoma skin cancer (NMSC) is the most prevalent cancer in humans and the incidence of NMSC is increasing in parallel with the aging of the population worldwide [1,2]. The most important risk factor for basal cell carcinoma (BCC) is the inability to tan, whereas cumulative ultraviolet (UV) exposure and age are the main risk factors for squamous cell carcinoma (SCC) [3,4]. Other environmental exposures such as ionizing radiation, immunosuppressive state and photosensitizing drugs including thiazide diuretics can increase the development of NMSCs. Also, medication-induced phototoxicity, in which drugs interact with UV radiation to cause cellular damage in the skin, may increase the carcinogenic potential of sun exposure [4–6].

Hydrochlorothiazide (HCTZ) is one of the most commonly prescribed diuretic and antihypertensive agents and it is also the first-line treatment in hypertensive disorder [7]. HCTZ exhibits known photosensitizing properties and it has been postulated that photocarcinogenic potential of thiazides may originate from UV radiation-induced transformation into potent mutagens [8,9]. HCTZ has previously been associated with increased risk of BCC and SCC [1,4,3]. In recent years, significantly increased risk of NMSC with HCTZ use was shown with a clear dose-dependent relationship [1,10]. The International Agency for Research of Cancer classified HCTZ as ‘possibly carcinogenic to humans’ in 2013 [11]. The Pharmacovigilance Risk Assessment Committee of the European Medicines Agency concluded that there was a causal relationship between HCTZ and NMSC development in 2018 [12].

Relapse risk of NMSC is influenced by several factors including tumor size and localization (H zone), histological subtype, perineural invasion, presence of immunodeficiency, and prior recurrence. While recent studies indicate that HCTZ use increases NMSC risk, it is unknown whether local recurrence (LR) is associated with HCTZ use. Therefore, we aimed to examine the association between HCTZ use and risk of NMSC LR.

## 2. Materials and methods

### 2.1. Study design and data collection

This study was an institutional review board-approved investigation (Approval number: KA21/363). We performed a retrospective case-control analysis based on the database of the university hospital. Patients with the following surgery codes, between January 1, 2013, and December 31, 2019, were identified from the university archives: ‘biopsy’, ‘malignant skin tumor excision’, ‘malignant skin tumor excision and skin grafting’, and ‘malignant skin tumor excision and flap reconstruction’. A total of 622 patients were found. Among these, those with benign tumors (n = 253), melanoma (n = 46), and skin appendage tumors (n = 8) were excluded. Fifteen patients with less than 6 months of follow-up were excluded. Finally, a total of 300 patients and 413 lesions were included in the study. The patients were divided into groups according to the pathological diagnosis, HCTZ use, and LR (Figure). Data collection included age, sex, location, size, pathological features of the lesions, recurrences, comorbidities, and follow-up time. Locations of NMSC cases were categorized as high-, medium-, and low (HML)-risk areas according to the National Comprehensive Cancer Network (NCCN) guideline. The size of lesions was grouped as follows: ≤10 mm, 10–20 mm, >20 mm. Hypertension (HT), diabetes mellitus (DM), hemodialysis (HD), kidney transplantation (KT), and liver transplantation (LTX) were selected as comorbidities.

### 2.2. Assessment of exposure

‘HCTZ-users’ were defined as having filled one or more prescriptions for an HCTZ-containing drug, and ‘non-HCTZ users’ were defined as patients never prescribed HCTZ and/or HCTZ containing drug during the follow-up period. A tumor was defined as recurrent if it was in the exact same location and had histopathologic findings consistent with the primary tumor.

### 2.3. Statistical analysis

Stata/MP14.1 (Stata Corp. Stata Statistical Software: Release 14) was used for descriptive and inferential analyses. The Shapiro–Wilk test was used to assess normality of distribution. Mean with standard deviation (SD) values were used to present normally distributed data. Chi-square tests were used for analysis of categorical outcomes. The Mann–Whitney *U* test was performed to compare nonparametric data between groups. Multivariable logistic regression analysis was performed separately to assess factors associated with LR in the BCC and SCC groups. A p-value less than 0.05 was accepted as significant.

## 3. Results

One hundred ninety-nine patients with 273 BCC lesions and 101 patients with 140 SCC lesions were included in the study. HCTZ had been used by 37 and 18 patients in the BCC and SCC groups, respectively. Recurrent cases were shown in Figure. The characteristics of study subjects according to the types of skin cancer and HCTZ use are shown in Table 1. LR of BCC was significantly more frequent in HCTZ users compared to nonusers (p = 0.010) (Table 1). Detailed demographical and clinical data of BCC and SCC patients are summarized in Tables 2 and 3, respectively.

In recurrent cases, the mean age of those with HCTZ use was significantly lower compared to those without HCTZ use (p = 0.048). BCC recurrences were associated with longer follow-up, and positive margins after excision in HCTZ users (p = 0.020 and 0.003, respectively). In BCC cases without HCTZ use, LR was associated with longer follow-up, lymphovascular invasion and perineural invasion (p < 0.001, p < 0.001, and p = 0.022, respectively).

In SCC cases with non-HCTZ use, frequency of LR was associated with longer follow-up, previous diagnosis of NMSC, and positive margins (p = 0.009, p < 0.001, and p = 0.031, respectively). In multivariable analysis for BCC cases, HCTZ use and longer follow-up were found to be independently associated with LR (p = 0.025 and p < 0.001, respectively), whereas, in SCC cases, previous diagnosis of NMSC and longer follow-up were independently associated with LR (p = 0.014 and p = 0.027, respectively) (Table 4). No recurrences have been observed in SCC cases with positive lymphovascular or perineural invasion (Table 3). Therefore, the variables, lymphovascular or perineural invasion, have been omitted in Table 4.

## 4. Discussion

This study aimed to evaluate the effect of HCTZ use on LR of NMSC, and, to the best of our knowledge, this is the first study addressing possible relationships between LR and HCTZ use in patients with NMSC. In this study, we found significantly increased risk of LR of BCC in patients who had received HCTZ.

The LR of BCC following primary excision is multifactorial and depends on patient characteristics, pathological features, and treatment factors. Previous studies have suggested an increased risk for LR in the elderly population [13,14]. The effect of increasing age may be associated with prolonged UV radiation exposure, cumulative photosensitizing effect of HCTZ, and diminished immunological status. Indeed, in our study, patients of the recurrent cases of BCC were younger than those of the group without LR. This may be due to the fact that there were seven cases of tumoral LR among HCTZ users.

The development of LR in HCTZ users among patients with BCC was found to be associated with longer follow-up and positive margins after excision. In the literature, LR frequency was reported to be 5%–14% following clear excisional margins, while the presence of positive margins after excision resulted in greater frequency of LR (10%–67%) [15]. In our study, LR was observed in 4% of BCC cases, and positive margins were independently associated with LR, indicating similarities with prior studies. It is established that 66% of BCC relapses are identified within 3 years, and 75% of LRs will occur within 5 years [15]. Therefore, longer follow-up is evidently associated with higher probability of detecting LR. LR also increased in non-HCTZ users with pathological features such as lymphovascular or perineural invasion. The majority of the previous research emphasizes that tumors with more aggressive histological subtypes and patients’ immunosuppression status were associated with higher LR [15,16]. Because of the small number of patients with LR, we could not perform subgroup evaluations based on histological subtype. Furthermore, due to the retrospective design of our study, we were unable to assess the presence, duration or degree of immunosuppression in our study.

Similar to previous reports, LR rate in our SCC group was 1.2% (18/140) [17]. Although nonsignificant, LR of SCC was more frequent in HCTZ users than nonusers (16.7% vs 11.8%). The small sample size of patients with SCC was possibly one of the factors that resulted in nonsignificance. A study with a larger sample size could provide different results. However, similar to previous reports, LR among SCC patients in those with HCTZ use was associated with positive margins, previous diagnosis of NMSC, and longer follow-up [17,18]. As discussed before, these are well-known risk factors for LR of SCC.

Our study has several limitations. Firstly, our sample size was smaller compared to other recent studies, especially those in which the population was selected nationwide. However, population-based studies did not include pathological features of lesions and LRs. We recognize that the sample size of our study may not have been large enough to detect an association between SCC LRs and HCTZ use. Second, information bias should be kept in mind since this is a retrospective case-control study, and, given the retrospective nature, we were unable to evaluate various parameters such as Fitzpatrick skin type, UV exposure, smoking status, and potential confounding medications of the patients in this study. Nonetheless, this study should shed light on the topic as it is the first one investigating HCTZ’s effect on LR. Lastly, we were not able to detect clear cumulative dose and rate of LR among NMSC due to the retrospective design of our study.

Protection from the sun may not be the only way to prevent LR in patients with NMSC who were previously treated with HCTZ. Considering the fact that thiazides are one of the key agents in controlling blood pressure and reducing the risk of cardiac ischemia and stroke, we would like to draw attention on their use in patients with a medical history of NMSC. Further studies with more participants on the subject should thoroughly evaluate the possible effect of HCTZ on NMSC recurrence. The risk/benefit ratio of using HCTZ in patients with a history of NMSC needs to be carefully considered. Considering limited patient follow-up and the fact that retrospective data could be conflicting, studies employing multicentered, double-blind, randomized controlled designs might be needed to conclusively determine whether HCTZ should not be used in patients previously diagnosed with NMSC.

## Figures and Tables

**Figure:**
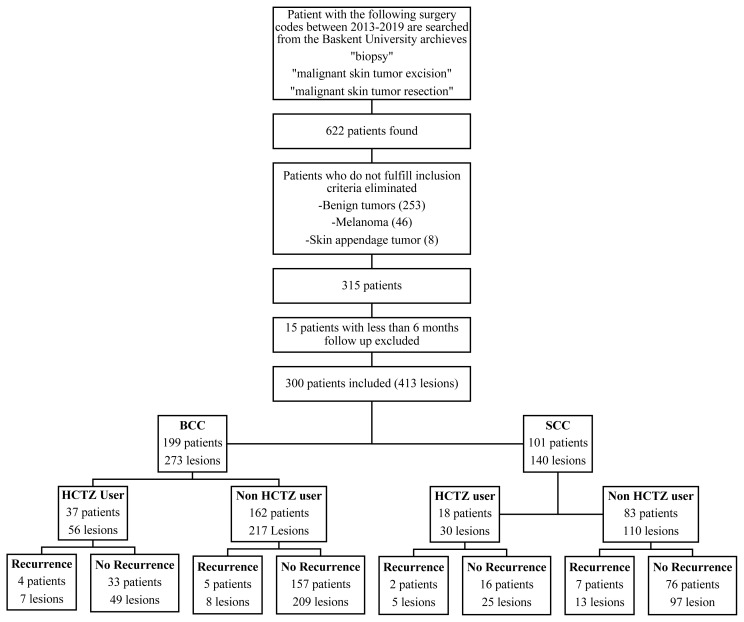
Patient selection and grouping according to the pathological diagnosis, HCTZ use, and LR.

**Table 1:** Characteristics of study subjects according to the types of skin cancer.

Characteristics	BCC			SCC		

	HCTZ (−)	HCTZ (+)	p-value	HCTZ (−)	HCTZ (+)	p-value

**Age**						
**Median (IQR)**	69.9 (12.1)	77.1 (8.8)	**<0.001**	71.2 (13.0)	80.1 (10.6)	**<0.001**
**Min–max**	33–97	63–92		40–95	38–97	

**Lesions**						
- **Male**	130 (%59.9)	31 (%55.4)	0.53	81 (%73.6)	19 (%63.3)	0.26
- **Female**	87 (%40.1)	25 (%44.6)		29 (%26.4)	11 (%36.7)	

**Tumor location**						
- **High risk**	120 (%55.3)	27 (%48.2)	0.07	56 (%50.9)	10 (%33.3)	0.14
- **Medium risk**	75 (%34.6)	17 (%30.4)		44 (%40.0)	18 (%60.0)	
- **Low risk**	22 (%10.1)	12 (%21.4)		10 (%9.1)	2 (%6.7)	

**Size (mm)**						
**<10**	138 (%63.6)	34 (%60.7)	0.65	65 (%59.1)	18 (%60.0)	0.55
**10–20**	58 (%26.7)	18 (%32.1)		26 (%23.6)	9 (%30.0)	
**>20**	21 (%9.7)	4 (%7.1)		19 (%17.3)	3 (%10.0)	

**Recurrence**	8 (%3.7)	7 (%12.5)	**0.01**	13 (%11.8)	5 (%16.7)	0.48

**Comorbidities**						
**HT**	102 (%47.0)	56 (%100.0)	**<0.001**	63 (%57.3)	30 (%100.0)	**<0.001**
**DM**	24 (%11.1)	11 (%11.6)	0.08	29 (%26.4)	4 (%13.3)	0.13
**Dialysis**	10 (%4.6)	2 (%3.6)	0.73	5 (%4.5)	1 (%3.3)	0.77
**Renal TX**	6 (%2.8)	2 (%3.6)	0.75	10 (%9.1)	1 (%3.3)	0.29
**Liver TX**	1 (%0.5)	0 (%0.0)	0.61	1 (%0.9)	0 (%0.0)	0.60

Values are presented as n (%) unless otherwise stated.

BCC, basal cell carcinoma; Bx, biopsy; DM, diabetes mellitus; HCTZ, Hydrochlorothiazide; IQR, interquartile range; NMSC, nonmelanoma skin cancer; SCC, squamous cell carcinoma; TX, transplantation.

**Table 2:** The association of recurrence and HCTZ use in BCC cases.

		NON HCTZ USER				HCTZ USER			
		
BCC		TOTAL	Recurrence (−)	Recurrence (+)	p	TOTAL	Recurrence (−)	Recurrence (+)	p

**Sex**									
Male		130 (%59.9)	126 (%96.9)	4 (%3.1)	0.56	31 (%55.4)	26 (%53.1)	5 (%71.4)	0.36
Female		87 (%40.1)	83 (%95.4)	4 (%4.6)		25 (%44.6)	23 (%46.9)	2 (%28.6)	

**Age**	Median (IQR)	69.9 (12.1)	70.0 (12.1)	64.8 (12.2)	0.18	77.1 (8.9)	78.0 (8.6)	71.4 (9.2)	**0,04**
	Min–max	33–97	33–97	49–87		63–92	63–92	63–85	

**Follow-up, days**	Mean (SD). days	369.6 (528.6)	329.7 (456.8)	1413.7 (1061.8)	**<0.001**	571.2 (638.2)	492.6 (588.3)	1121.4 (749.2)	**0.02**
	Min–max	180–2850	180–2650	180–2850		180–2160	180–2160	180–1980	

**Previous NMSC**		34 (%15.7)	32 (%15.3)	2 (%25.0)	0.45	15 (%26.8)	14 (%28.6)	1 (%14.3)	0.42

**Size (mm)**									
≤10		138 (%63.6)	132 (%63.2)	6 (%75.0)	0.64	34 (%60.7)	29 (%59.2)	5 (%71.4)	0.68
10–20		58 (%26.7)	57 (%27.3)	1 (%12.5)		18 (%32.1)	16 (%32.6)	2 (%28.6)	
>20		21 (%9.7)	20 (%9.6)	1 (%12.5)		4 (%7.1)	4 (%8.2)	-	

**Tumor location**									
High risk		120 (%55.3)	115 (%55.0)	5 (%62.5)	0.62	27 (%48.2)	23 (%46.9)	4 (%57.1)	0.11
Medium risk		75 (%34.6)	72 (%34.5)	3 (%37.5)		17 (%30.4)	17 (%34.7)	0 (−)	
Lower risk		22 (%10.1)	22 (%10.5)	-		12 (%21.4)	9 (%18.4)	3 (%42.9)	

**BCC subtypes**									
Nodular		131 (%60.4)	127 (%60.8)	4 (%50.0)	0.96	33 (%58.9)	28 (%57.1)	5 (%71.4)	0.19
Superficial		34 (%15.7)	33 (%15.8)	1 (%12.5)		14 (%25.0)	13 (%26.5)	1 (%14.3)	
Basosquamous		23 (%10.6)	22 (%10.5)	1 (%12.5)		5 (%8.9)	5 (%10.2)	0 (−)	
İnfiltrative		16 (%7.4)	14 (%6.7)	2 (%25.0)		1 (%1.8)	0 (−)	1 (%14.3)	
Adenoid		3 (%1.4)	3 (%1.4)	-		0 (−)	0 (−)	0 (−)	
Nodulocystic		3 (%1.4)	3 (%1.4)	-		1 (%1.8)	1 (%2.0)	0 (−)	
Fibroepithelial		2 (%0.9)	2 (%1)	-		0 (−)	0 (−)	0 (−)	
İnfundibulocystic		1 (%0.4)	1 (%0.4)	-		0 (−)	0 (−)	0 (−)	
Metatypical		1 (%0.4)	1 (%0.4)	-		1 (%1.8)	1 (%2)	0 (−)	
Micronodular		1 (%0.4)	1 (%0.4)	-		1 (%1.8)	1 (%2)	0 (−)	
Sclerosing		1 (%0.4)	1 (%0.4)	-		0 (−)	0 (−)	0 (−)	

**Ulceration**		137 (%63.1)	132 (%63.2)	5 (%62.5)	0.97	39 (%69.6)	33 (%67.4)	6 (%85.7)	0.32

**Lymphovascular invasion**		2 (%0.9)	1 (%0.5)	1 (%12.5)	**<0.001**	0 (%0)	0 (%0)	0 (%0)	-

**Perineural invasion**		4 (%1.8)	3 (%1.4)	1 (%12.5)	**0.02**	0 (%0)	0 (%0)	0 (%0)	-

**Positive margin**		30 (%13.8)	28 (%13.4)	2 (%25.0)	0.35	6 (%10.7)	3 (%6.1)	3 (%42.9)	**0.003**

**Treatment**									
Excisional bx		48 (%22.1)	44 (%21.1)	4 (%50.0)	0.13	13 (%23.2)	11 (%22.5)	2 (%28.6)	0.09
Bx and skin graft		67 (%30.9)	66 (%31.6)	1 (%12.5)		20 (%41.1)	20 (%40.8)	0 (%0)	
Bx and flap		102 (%47.0)	99 (%47.3)	3 (%37.5)		23 (%35.7)	18 (%36.7)	5 (%71.4)	

BCC, basal cell carcinoma; BX, biopsy; DM, diabetes mellitus; HCTZ, hydrochlorothiazide; NMSC, nonmelanoma skin cancer, SD; standard deviation.

**Table 3:** The association of recurrence and HCTZ use in SCC cases.

		NON HCTZ USER				HCTZ USER			
		
SCC		Total	Recurrence (−)	Recurrence (+)	p	Total	Recurrence (−)	Recurrence (+)	p

**Sex**									
Male		81 (%73.6)	70 (%72.2)	11 (%84.6)	0.33	19 (%63.3)	16 (%64.0)	3 (%60.0)	0.86
Female		29 (%26.4)	27 (%27.8)	2 (%15.4)		11 (%36.7)	9 (%36.0)	2 (%40.0)	

**Age**	Median (IQR)	71.2 (13.0)	70.9 (13.0)	73.2 (13.7)	0.57	80.1(10.6)	80.0 (11.7)	80.2 (1.3)	0.80
	Min–max	40–95	40–95	44–90		38–97	38–97	79–82	

**Follow up**	Mean (SD)	503.0 (602.5)	432.6 (519.9)	1027.6 (892.7)	**0.009**	350 (371.4)	307.2 (326.2)	564 (540.9)	0.35
	Min–max	180–2430	180–2430	180–2200		180–1530	180–1530	180–1320	

**Previous NMSC**		31 (%28.2)	22 (%22.7)	4 (%30.8)	**<0.001**	11 (%36.7)	8 (%32.0)	3 (%60.0)	0.23

**Size (mm)**									
<10		65 (%59.1)	58 (%59.8)	7 (%53.8)	0.83	34 (%60.7)	29 (%59.2)	3 (%60.0)	0.67
10–20		26 (%23.6)	23 (%23.7)	3 (%23.1)		18 (%32.1)	16 (%32.6)	2 (%40.0)	
>20		19 (%17.3)	16 (%16.5)	3 (%23.1)		4 (%7.1)	4 (%8.2)	0 (%0)	

**Tumor location**									
High risk		56 (%50.9)	52 (%53.6)	4(%30.8)	0.23	10 (%33.3)	7 (%28.0)	3 (%60.0)	0.35
Medium risk		44 (%40.0)	36 (%37.1)	8 (%61.5)		18 (%60.0)	16 (%64.0)	2 (%40.0)	
Lower risk		10 (%9.1)	9 (%9.3)	1 (%7.7)		2 (%6.7)	2 (%8.0)	0 (%0)	

**SCC subtypes**									
In situ		10 (%9.1)	10 (%10.3)	0	0.36	5 (%16.7)	5 (%20.0)	0	0.39
Well-differentiated		80 (%72.3)	70 (%72.2)	10 (%76.9)		20 (%66.7)	15 (%60.0)	5 (%100)	
Moderately differentiated		17 (%15.5)	15 (%15.5)	2 (%15.4)		0 (%0)	0 (%0)	0	
Poorly differentiated		2 (%1.8)	1 (%1.0)	1 (%7.7)		4 (%13.3)	4 (%16.0)	0	
Adenosquamous		1 (%0.9)	1 (%1.0)	0 (%0)		1 (%3.3)	1 (%4.0)	0	

**Ulceration**		40 (%36.4)	33 (%34.0)	7 (%53.9)	0.16	11 (%36.7)	9 (%36.0)	2 (%40.0)	0.86

**Lymphovascular invasion**		4 (%3.6)	4 (%4.1)	0 (%0)	0.45	2 (%6.7)	2 (%8.0)	0 (%0)	0.51

**Perineural invasion**		2 (%1.8)	2 (%2.1)	0 (%0)	0.60	5 (%16.7)	5 (%20.0)	0 (%0)	0.27

**Positive margin**		19 (%17.3)	14 (%14.4)	5 (%38.5)	**0.03**	3 (%10.0)	2 (%8.0)	1(%20.0)	0.41

**Treatment**									
Excisional bx		35 (%31.8)	30 (%30.9)	5 (%38.5)	**0.03**	7 (%23.3)	7 (%28.0)	0 (%0)	0.19
Bx and skin graft		32 (%29.1)	25 (%25.8)	7 (%53.8)		9 (%30.0)	6 (%24.0)	3 (%60.0)	
Bx and flap		43 (%39.1)	42 (%43.3)	1 (%7.7)		14 (%46.7)	12 (%48.0)	2 (%40.0)	

Bx, biopsy; DM, diabetes mellitus; HCTZ, hydrochlorothiazide; IQR, interquartile range; NMSC, nonmelanoma skin cancer; SCC, squamous cell carcinoma; SD, standard deviation.

**Table 4:** Multivariable analysis of recurrence in BCC cases.

Recurrences in BCC	Odds ratio	Std. Err.	z	p > |z|	[95% Conf. interval]
**Age**	0.98	0.03	−0.53	0.59	0.92–1.04
**Sex**	1.08	0.72	0.12	0.90	0.29–4.05
**Size**	0.70	0.41	−0.59	0.55	0.22–2.24
**HML area**	0.82	0.31	−0.50	0.61	0.38–1.75
**Previous NMSC**	2.40	1.96	1.07	0.28	0.48–11.94
**HCTZ**	4.83	3.39	2.25	**0.02**	1.22–19.12
**Follow-up**	1.00	0.04	4.09	**<0.001**	1.01–1.02
**Ulceration**	0.28	0.22	−1.58	0.11	0.05–1.35
**Lymphvascular invasion**	0.08	0.17	−1.13	0.25	0.001–6.26
**Perineural invasion**	0.34	0.78	−0.47	0.63	0.003–30.3
**Positive margin**	2.98	2.39	1.36	0.17	0.617–14.38

**Table 5:** Multivariable analysis of recurrence in SCC cases.

Recurrences in SCC	Odds ratio	Std. Err.	z	p > |z|	[95% Conf. interval]
**Age**	1.01	0.02	0.72	0.47	0.96–1.07
**Sex**	1.19	0.91	0.23	0.81	0.26–5.36
**Size**	1.25	0.57	0.51	0.61	0.5–3.06
**HML area**	1.20	0.39	0.56	0.57	0.63–2.27
**Previous NMSC**	0.21	0.13	−2.46	**0.01**	0.06–0.73
**HCTZ**	2.14	1.56	1.04	0.29	0.51–8.98
**Follow-up**	1.00	0.00	2.21	**0.02**	1.00–1.00
**Ulceration**	0.49	0.33	−1.05	0.29	0.13–1.83
**Positive margin**	2.73	1.93	1.42	0.15	0.68–10.95

BCC: basal cell carcinoma; HCTZ: Hydrochlorothiazide; HML area: High-, medium-, low-risk area; NMSC: nonmelanoma skin cancer; SCC: squamous cell carcinoma.
